# Auditory Neuropathy Spectrum Disorder (ANSD)—Clinical Characteristics and Pathogenic Variant Analysis of Three Nonsyndromic Deafness Families

**DOI:** 10.1155/2020/8843539

**Published:** 2020-12-22

**Authors:** Rongqun Zhai, Haifeng Feng, Qingli Li, Wei Lu, Danhua Liu, Yongan Tian, Huanfei Liu, Ruijun Li, Bin Zuo, Wenxue Tang, Hongen Xu, Bei Chen

**Affiliations:** ^1^Department of Otorhinolaryngology, Head and Neck Surgery, The First Affiliated Hospital of Zhengzhou University, Zhengzhou 450052, China; ^2^Department of Pediatrics, The Third Affiliated Hospital of Zhengzhou University, Zhengzhou 450052, China; ^3^Center for Applied Precision Medicine, The Second Affiliated Hospital of Zhengzhou University, Zhengzhou 450014, China; ^4^BGI College, Zhengzhou University, Zhengzhou 450052, China; ^5^Henan Institute of Medical and Pharmaceutical Sciences, Zhengzhou University, Zhengzhou 450052, China; ^6^Precision Medicine Center, Academy of Medical Science, Zhengzhou University, Zhengzhou 450052, China

## Abstract

**Objective:**

To analyze the phenotypic features and pathogenic variants of three unrelated families presenting with nonsyndromic auditory neuropathy spectrum disorder (ANSD).

**Methods:**

Three recruited families that were affected by congenital deafness were clinically evaluated, including a detailed family history and audiological and radiological examination. The peripheral blood of all patients and their parents was collected for DNA extraction, and then, the exonic and flanking regions were enriched and sequenced using targeted capture and high-throughput sequencing technology. Bioinformatics analyses and the Sanger sequencing were carried out to screen and validate candidate pathogenic variants. The pathogenicity of candidate variants was evaluated by an approach that was based on the standards and guidelines for interpreting genetic variants as proposed by the American College of Medical Genetics and Genomics (ACMG).

**Results:**

Four patients in three families were diagnosed as nonsyndromic ANSD, and all exhibited *OTOF* gene mutations. Among them, two individuals in family 1 (i.e., fam 1-II-2 and fam 1-II-3) carried homozygous variants c.[2688del];[2688del] (NM_194248.3). Two individuals from family 2 (fam 2-II-1) and family 3 (fam 3-II-4) carried compound heterozygous variants c.[4960G>A];[1469C>G] and c.[2675A>G];[2977_2978del], respectively.

**Conclusions:**

Three unrelated pedigrees with ANSD were caused by pathogenic variants in the *OTOF* gene. Five mutations were found and included c.2688del, c.2675A>G, c.2977_2978del, c.4960G>A, and c.1469C>G, of which the first two are novel and expanded mutational spectrum of the *OTOF* gene, thus having important implications for genetic counseling of the family.

## 1. Introduction

Hereditary hearing impairment is a frequently observed sensory and disabling disease, which causes incredibly negative consequences to patients—both psychologically and physiologically. Hereditary hearing impairment can be divided into two distinct categories that include the syndromic type, with abnormalities found in other parts of the body, and the nonsyndromic type. Nonsyndromic hearing loss (NSHL) accounts for 70% of the overall incidence and is an exceedingly heterogeneous disease [1]. NSHL is mainly transmitted as autosomal recessive, autosomal dominant, or X-linked inheritance patterns. Hitherto, up to 100 genes have been implicated in hereditary nonsyndromic deafness (https://hereditaryhearingloss.org/).

Auditory neuropathy (AN), which is a particular type of auditory dysfunction with impaired speech comprehension, was first coined and nominated by Sttar in 1996 [2], which is also referred to as an auditory neuropathy spectrum disorder (ANSD). ANSD is subdivided into “postsynaptic” or “presynaptic” types, depending on whether the auditory nerve is involved or the inner hair cells (IHC), and synaptic lesions are involved [3]. ASND can cause varying degrees of hearing impairment. Diagnosis can be made on the presentation of severe anomalies or absence in the auditory brainstem response (ABR), and the presence of otoacoustic emissions (OAEs) and cochlear microphonics (CM), as well as an attenuation in speech perception that is out of proportion to the pure-tone threshold [2, 4]. We have arrived at a plausible conjecture that the auditory pathway up to and including the outer hair cells (OHCs) in ANSD patients is intact and that the primary lesions might represent a malfunction or defect of the IHCs, the auditory synapses, or the auditory nerve itself [2]. These lesions can affect the processing ability of rapid acoustic signals so that the sound signals cannot be transmitted synchronously from the inner ear to the auditory cortex. The etiology of ANSD is complex, and various etiologies have been found, of which about 40 percent can be accounted for by genetic origin [4–6]. Recently, owing to advances in next-generation sequencing, genetic etiologies underlying ASND, which include *OTOF*, *DFNB59*, and *DIAPH3* genes, have been frequently revealed [7]. To date, nearly 200 variants of the *OTOF* gene have been deposited in the Human Gene Mutation Database (HGMD), which is the primary causative gene in infants affected by ASND.

The *OTOF* gene is located at the DFNB9 locus and was the first identified causative gene associated with nonsyndromic ANSD by Yasunage et al. in 1999 [8]. The *OTOF* gene is located in 2p23.1 and contains 48 exons that encode the otoferlin protein [9]. The coding product has multiple isoforms with unequal lengths due to alternative translation start sites and splicing sequences. Among them, a long isoform with 1997 amino acid residues contains a C-terminal transmembrane domain (TMD) that is involved in docking to the cytoplasmic membrane and six C2 domains (C2A-C2F) that permit binding to Ca^2+^ and Ca^2+^-dependent related proteins [10]. Studies have shown that the otoferlin protein plays an essential role in the exocytosis and replenishment of neurotransmitters in IHC synaptic vesicles and does so by triggering synaptic membrane fusion in a Ca^2+^-activated manner [10–13]. It has also been demonstrated that the otoferlin protein is invariably expressed and concentrated in the basolateral region of the IHCs in the mature mouse cochlea and is an essential component of the presynaptic structure of IHCs [14]. Thus, researchers speculated that mutations in *OTOF* might affect the structure or function of the otoferlin protein at the IHC ribbon synapse, leading to differential magnitudes in hearing loss. Patients with mutations in *OTOF* exhibit presynaptic nonsyndromic AN; moreover, cochlear implantation (CI) can achieve a favorable outcome [15–17].

Herein, we examined and described in some detail patients affected by ANSD; following which, we performed whole-exome and the Sanger sequencing to unravel possible etiologies in these sporadic families with ANSD. Consequently, mutations in the *OTOF* gene were identified as being disease-causing in these patients.

## 2. Materials and Methods

### 2.1. Family Description and Clinical Examination

In this study, families 1-3, which comprised three unrelated Chinese pedigrees, including four affected patients with nonsyndromic hearing loss (NSHL) and their phenotypically healthy parents, were recruited. Four affected offspring who presented with prelingual and bilateral sensorineural deafness were diagnosed by head and neck surgery at the First Affiliated Hospital of Zhengzhou University. Detailed family and medical histories were recorded. Subsequently, all patients underwent speech audiometry, electrootoscopy, cochlear microphonic (CM), otoacoustic emission (OAE), auditory brainstem response (ABR), and multifrequency steady-state auditory evoked response (ASSR). In addition, other audiological examinations were performed to evaluate audiological characteristics. Temporal bone CT and magnetic resonance imaging (MRI) were also carried out to exclude inner ear dysplasia and intracranial lesions. Peripheral blood was taken after obtaining signed informed consent from all participants. The experimental protocol of this study was authorized by the local Medical Ethics Committee of the First Affiliated Hospital of Zhengzhou University and was compliant with the Helsinki Declaration of the World Medical Association.

### 2.2. Whole-Exome Sequencing

All four patients (3 probands and an affected sib) in our study were subjected to a preliminary screening using whole-exome sequencing. DNA extraction, fragmentation, library construction, targeted enrichment, and sequencing were performed as described previously [18].

### 2.3. Bioinformatics Analysis and Variation Interpretation

Sequencing fragments were processed using Trimmomatic [19] to eliminate adapters and inferior reads. After quality control, the clean reads were aligned to the human reference genome (version number hg19) using the Burrows-Wheeler Aligner [20]. The GATK HaplotypeCaller software [21] (version 4.1.2) was also applied to detect single-base variations and short insertions or deletions. Variant annotation, filtering, and interpretation were carried out as described previously [18].

### 2.4. Sanger Sequencing

The candidate mutations were validated by PCR amplification and the Sanger sequencing. The primers described below (Table 1) were designed and synthesized with the aim of detecting variations in probands and their families. PCR amplification was carried out using a 2x Taq Master Mix kit with a system of 25 *μ*L. The amplified products were identified by 2.2% agarose gel electrophoresis to determine the fragment size and then purified with a DNA product purification kit. Purified PCR products were sequenced using the SeqStudio Genetic Analyzer (Applied Biosystems/Life Technologies, Carlsbad, CA, USA).

## 3. Results

### 3.1. Clinical Features

**Figure 1:**
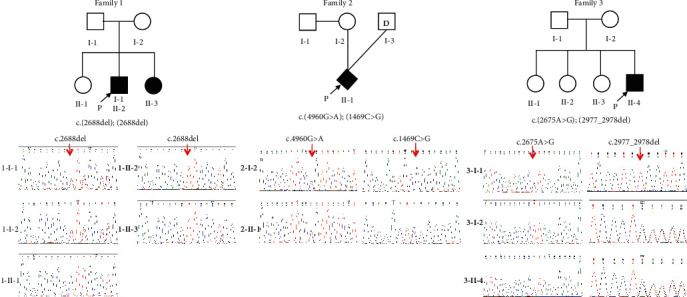
Pedigrees of families and the sequence analysis of the *OTOF* mutations in their family members. Blackened symbols: affected individuals.

The comprehensive family and medical histories revealed that all families had no similar cases or exposure to risk factors, such as neonatal jaundice, and infections. Three pedigrees are plotted in Figure 1. The results of electrootoscopy showed that four individuals (i.e., 1-II-2, 1-II-3, 2-II-1, and 3-II-4) displayed intact bilateral tympanic membranes, and there was evidence of an apparent bilateral hydrotympanum in the proband 1-II-2. ABRs were indistinguishable or absent in all patients, and their ASSR examination also showed severe-to-profound sensorineural deafness in both ears. Normal bilateral OAEs and CM were elicited in three patients (i.e., 1-II-3, 2-II-1, and 3-II-4). Neither ear of proband 1-II-2 passed the OAE test as compared with the others; however, both ears exhibited CM. Neither dysplasias of the inner or middle ear nor intracranial lesions were found by CT and MRI in any of the affected children. Furthermore, four affected offspring with only isolated hearing loss were unaccompanied by any other abnormality. Combined with the above characteristics (Table 2), the affected offspring were diagnosed as nonsyndromic auditory neuropathy. Among the four patients with ANSD, only the proband 1-II-2 and his sister (1-II-3) underwent cochlear implantation in the Department of Otolaryngology at the First Affiliated Hospital of Zhengzhou University, China, in late 2018 and 2019, respectively.

### 3.2. Whole-Exome Sequencing Identified Candidate Mutations in the *OTOF* Gene

All patients underwent whole-exome sequencing, yielding 12.3 Gbps, 12.0 Gbps, 17.1 Gbps, and 14.2 Gbps of raw data, respectively. More than 99.6% of all raw data was mapped to the human reference genome sequence. The average sequencing depth of the targeted regions was ≥100X, and covered ≥95% of the targeted regions.

We then filtered out variants with an allelic frequency > 0.1% in the gnomAD database and kept variants in the known deafness genes (https://hereditaryhearingloss.org/). Homozygous (c.[2688del];[2688del]) and compound heterozygous damaging variants (i.e., c.[4960G>A];[1469C>G] and c.[2675A>G];[2977_2978del]) were found in probands 1-II-2, 2-II-1, and 3-II-4, respectively. All mutations of the *OTOF* gene were verified in family members by the Sanger sequencing, which showed that their parents were all heterozygous carriers.

### 3.3. Variation Interpretation

#### 3.3.1. c.2688del in Family 1

The four-year-old proband I-II-2 and his two-year-old sister all carried the c.2688del variant in a homozygous state. They inherited the damaging mutations from both their heterozygous healthy parents. The c.2688del variant is a frameshift mutation causing a loss of function (LOF) of the *OTOF* gene and LOF is a known mechanism of disease for this gene (PVS1). This novel mutation was absent in 1000 Genomes, ExAC, and gnomAD (PM2). Multiple lines of computational evidence supported a deleterious effect of G nucleotide deletion at position 2688 on the *OTOF*gene (PP3) . The clinical manifestations of the patients were consistent with the phenotype of the disease caused by the *OTOF* gene (PP4). Thus, based on ACMG standards and guidelines for interpreting sequencing variants [22], the variant c.2688del was classified as pathogenic (Table 3).

#### 3.3.2. Compound Heterozygous Variants c.[4960G>A];[1469C>G] in Family 2

The two-year-old proband 2-II-1, born an artificially inseminated child, was found to carry compound heterozygous variants referred to as c.[4960G>A];[1469C>G]. Of these, c.4960G>A was from the mother, and c.1469C>G was from a sperm donor or occurred *de novo*. The c.4960G is the final base before the splice site, while G-to-A substitution at the position leads to the alteration of the splicing process, which is a known mechanism of disease (PVS1). The minor allelic frequencies of c.4960G>A were very low in 1000 Genomes, ExAC, and gnomAD (PM2), and the mutation was expected to result in aberrant splicing according to prediction softwares (PP3). The clinical manifestations of the patients were consistent with the phenotype of the disease known to be caused by the *OTOF* gene damaging mutations (PP4). The mutation c.1469C>G (p.Pro490Arg) was rare in the normal population database (PM2). In an Omani family with auditory neuropathy, the five affected children were all homozygous for the p.Pro490Arg mutations [23] (PM3). Computational softwares supported the pathogenicity of c.1469C>G (PP3). The clinical manifestations of the patients were consistent with the disease phenotype caused by the *OTOF* gene (PP4). Thus, according to ACMG standards and guidelines, the variants c.4960G>A and c.1469C>G were classified as pathogenic and likely pathogenic, respectively (Table 3).

#### 3.3.3. Compound Heterozygous Variants c.[2675A>G];[2977_2978del] in Family 3

The compound heterozygous variants c.[2675A>G];[2977_2978del] were detected in proband 3-II-4. Among them, the reported mutation of c.2977_2978del was transmitted from the mother and was a frameshift mutation [24]. It causes LOF of the *OTOF* gene, which is a known mechanism of disease (PVS1). The frequency of mutation c.2977_2978del was very low in 1000 Genomes, ExAC, and gnomAD (PM2). Multiple prediction softwares support the pathogenicity of this variant (PP3). The patient's clinical phenotype was highly specific for the *OTOF* gene (PP4). In addition, the c.2675A>G was from the father, was a missense mutation, and was not found in public population databases, including 1000 Genomes, ExAC, and gnomAD (PM2). There was a mutation in trans of c.2675A>G, which is known to be pathogenic (PM3), and c.2675A>G was expected to yield aberrant splicing according to the prediction softwares (PP3). The patient's clinical phenotype was highly specific for the *OTOF* gene (PP4). Thus, the variant c.2977_2978del was classified as pathogenic, and the variant c.2675A>G was categorized as likely pathogenic (Table 3).

## 4. Discussion

ANSD presents markedly genotypic and phenotypic heterogeneity. However, ANSD explained by mutations in the *OTOF* gene has been proven to be primarily interrelated with congenital, severe-to-profound, nonsyndromic sensorineural deafness [25]. Herein, our concern with regard to patients affected by congenital deafness, whose clinical and audiological characteristics matched the above phenotype, also met the diagnostic criteria for ANSD (Table 2). Furthermore, bioinformatics analysis confirmed that the three families exhibited two novel mutations (i.e., c.2688del, c.2675A>G) and three reported variations (i.e., c.4960G>A, c.2977_2978del and c.1469C>G) in the *OTOF* gene [23, 24, 26]. Combining the verification by the Sanger sequencing in the corresponding parents and siblings (Table 3), variants c.[2688del];[2688del], c.[4960G>A];[1469C>G], and c.[2675A>G];[2977_2978del] were confirmed to be likely pathogenic/pathogenic for three families.

Mutations in the *OTOF* gene, especially those affecting highly conserved domains, display an increased abominable effect on the structure or function of the otoferlin protein. In this study, c.2688del (p.Lys896AsnfsTer104) and c.2977_2978del (p.Gln994ValfsTer7) represented two frameshift mutations. The novel p.Lys896AsnfsTer104 was found in the region between the C2C and C2D domains, while the p.Gln994ValfsTer7 that has been discussed in a familial case of temperature sensitive nonsyndromic auditory neuropathy (TS-NSRAN) was located in the functional domain C2D [24]. Both frameshift mutations (i.e., c.2688del and c.2977_2978del) were predicted to cause the translation process to terminate prematurely, causing the loss of the downstream domain or engendering a truncated otoferlin protein as a consequence. The three identified missense mutations c.4960G>A, c.1469C>G, and c.2675A>G cause transversion of highly conserved amino acids from glycine to serine (p.Gly1654Ser), proline to arginine (p.Pro490Arg), and lysine to arginine (p.Lys892Arg), respectively. Among them, the G>A nucleotide change at position 4960 was located in the exon/intron junction that was expected to result in aberrant splicing, thus causing an abnormal amino acid chain of the otoferlin protein. The reported missense mutation c.1469C>G was located in the relevant C2C domain and was confirmed to affect the function of protein products [23]. The mutation c.2675A>G was found in the region between the C2C and C2D domains and was predicted to result in deleterious splicing. In summary, we speculate that these variations that affect the protein structure or the capacity of proteins to bind Ca^2+^ might cause a reduction or deficiency of neurotransmitters in IHCs synaptic vesicles and the occurrence of the characteristic phenotype of ASND. However, further studies are warranted to elucidate the specific pathogenic molecular mechanism of these mutations.

Mutations in the *OTOF* gene cause presynaptic ANSD, with varying prevalence and hotspot mutations in different ethnic cohorts. For example, in Spanish population, the prevalence of nonsyndromic ANSD caused by mutations in the *OTOF* gene was estimated to be 87%, and the hotspot mutation is c.2485C>T (p.Gln829Ter) [27, 28], while the prevalence is 57% in the Japanese population, and the hotspot mutation is c.5816G>A (p.Arg1939Gln) [29, 30]. Similarly, this frequency was more than 41% in the mainland Chinese population [31], but no hotspot mutations were detected. Despite all of this, clinical management decisions for patients with ANSD lead to cochlear implantation (CI). The effect of CI varies according to the location of the lesion; however, presynaptic ANSD were found suitable for intervention, contrary to postsynaptic ANSD [32]. In this current study, two affected offspring harboring novel homozygous mutations c.2688del in the *OTOF* gene underwent CI and received acceptable results as expected. Their speech recognition ability was also effectively improved. Hence, prior to this invasive treatment, a precise molecular diagnosis would be helpful in the differential diagnosis and prognosis of patients with ANSD.

## 5. Conclusions

In conclusion, we confirmed that four individuals with ANSD (i.e., 1-II-2, 1-II-3, 2-II1, and 3-II-4) had mutations in the *OTOF* gene as shown by next-generation sequencing. The proband 1-II-2 and his sister 1-II-3 carried a homozygous variants c.[2688del];[2688del]. The compound heterozygous variants defined as c.[4960G>A];[1469C>G] and c.[2675A>G];[2977_2978del] were identified in probands 2-II-1 and 3-II-4, respectively. Convincing evidence supports the notion that these variations are causative factors of nonsyndromic ANSD in the three families. The discovery of c.2688del and c.2675A>G mutations expands the spectrum of mutations found in the *OTOF* gene and provides a new reference point for the genetic diagnosis of auditory neuropathy.

## Figures and Tables

**Table 1:** Primers used in the Sanger sequencing.

Variants	Primers (5' to3')
c.2688del	Forward: GGGTCCTCACTCACTGGTGTAGA Reverse: AGCTCTGACCAGGGCCTCT
c.4960G>A	Forward: GACCAGGTTTAGGCTGAGGACA Reverse: TCCCACAGACATGGCTACAATAT
c.1469C>G	Forward: TTCCTTCCCTTCAGGCCACT Reverse: CACCAGGGCAAGACTTCAGT
c.2977_2978del	Forward: TCCTTGTCGTCCCTGTCTTG Reverse: GGCTTCCAGGAGGTCAAGG
c.2675A>G	Forward: TTCCCATTCTTGGCTCTTCTCT Reverse: CACAGCATTCCCGACATCTT

**Table 2:** Genotype and phenotype of four individuals with ANSD in this study.

Subjects	Genotype	ABR	DPOAE	CM	ASSR	
					Right ear	Light ear
1-II-2	c.[2688del];[2688del]	Absent	Bil absent	Present	Profound	Profound
1-II-3	c.[2688del];[2688del]	Absent	Bil present	Present	Profound	Profound
2-II-1	c.[4960G>A];[1469C>G]	Absent	Bil present	Present	Severe	Severe
3-II-4	c.[2675A>G];[2977_2978del]	Absent	Bil present	Present	Profound	Profound

Abbreviations: ABR, auditory brainstem response; CM, cochlear microphonics; DPOAE, distortion product otoacoustic emissions; ASSR, multifrequency steady-state auditory evoked response; Bil, bilateral.

**Table 3:** Pathogenic variants in the *OTOF* gene identified in this study.

Proband	Nucleotide	Codon alteration	Type	Classification	ACMG evidence	References
1-II-2, 1-II-3	c.2688del	p.Lys896AsnfsTer104	Hom	P	PVS1, PM2, PP3, PP4	This study
2-II-1	c.4960G>A	p.Gly1654Ser	Het	P	PVS1, PM2, PP3, PP4	[26]
	c.1469C>G	p.Pro490Arg	Het	LP	PM2, PM3, PP3, PP4	[23]
3-II-4	c.2977_2978del	p.Gln994ValfsTer7	Het	P	PVS1, PM2, PP3, PP4	[24]
	c.2675A>G	p.Lys892Arg	Het	LP	PM2, PM3, PP3, PP4	This study

Abbreviations: Hom, homozygote; Het, heterozygote; P, pathogenic; LP, likely pathogenic; PM, pathogenic moderate; PP, pathogenic supporting; PVS1, pathogenic strong.

## Data Availability

The data that support the findings of this study are available from the corresponding authors upon reasonable request.
